# Efficacy and Tolerability of Bupropion in Major Depressive Disorder with Comorbid Anxiety Symptoms: A Systematic Review

**DOI:** 10.3390/ijms262411767

**Published:** 2025-12-05

**Authors:** Mario Pinzi, Alessandro Cuomo, Despoina Koukouna, Giacomo Gualtieri, Caterina Pierini, Maria Beatrice Rescalli, Simone Pardossi, Benjamin Patrizio, Andrea Fagiolini

**Affiliations:** Department of Molecular Medicine, School of Medicine, University of Siena, 53100 Siena, Italy; mario.pinzi@student.unisi.it (M.P.); alessandro.cuomo@unisi.it (A.C.); koukounadespoina@gmail.com (D.K.); giacomo.gualtieri2@unisi.it (G.G.); c.pierini@student.unisi.it (C.P.); m.rescalli@student.unisi.it (M.B.R.); s.pardossi@student.unisi.it (S.P.); benjamin.patrizio@student.unisi.it (B.P.)

**Keywords:** bupropion, major depressive disorder, anxious depression, comorbidity, systematic review

## Abstract

Anxiety symptoms are highly prevalent in major depressive disorder (MDD) and are associated with greater severity, functional impairment, and poorer treatment outcomes. Bupropion is widely used in clinical practice and is generally considered to have a favorable tolerability profile, but its effects on comorbid anxiety remain uncertain. We conducted a PRISMA-guided systematic review of randomized controlled trials, pooled analyses, and open-label comparative studies evaluating bupropion in adults with MDD and clinically significant anxiety symptoms. Searches of PubMed, Scopus and Web of Science were performed through August 2025. Outcomes included validated measures of anxiety and depressive symptoms and reported tolerability. Risk of bias was assessed using RoB 2 and ROBINS-I, and certainty of evidence was evaluated using GRADE. Six studies (n ≈ 3700) met inclusion criteria. Anxiety was a predefined secondary outcome in some trials and a post hoc or exploratory measure in others. Across designs, bupropion was generally associated with improvements in anxiety and depressive symptoms on secondary or exploratory anxiety measures. In pooled patient-level analyses, SSRIs showed a modest advantage over bupropion in patients with high baseline anxiety, whereas individual randomized and open-label studies found no significant between-group differences. None of the included studies reported a clear signal of anxiety worsening with bupropion on the anxiety measures used. Tolerability findings indicated a lower risk of sexual dysfunction with bupropion compared with SSRIs, while insomnia occurred more frequently but was generally manageable. Low-certainty evidence suggests that bupropion may provide clinically relevant improvement in anxiety symptoms in adults with MDD, with generally comparable efficacy to SSRIs in most presentations but a modest SSRI advantage in highly anxious subgroups. Interpretation should consider that anxiety outcomes were often secondary or exploratory and that several studies were at risk of bias. Well-designed randomized trials with anxiety as a primary endpoint are needed.

## 1. Introduction

Major depressive disorder (MDD) remains one of the leading causes of global disability and disease burden, and its course is frequently complicated by the presence of significant anxiety symptoms, resulting in the clinical construct of anxious depression. Epidemiological data suggest that between 40% and 60% of patients with MDD meet operational definitions of anxious depression, depending on criteria used, such as a Hamilton Depression Rating Scale (HAM-D) anxiety–somatization subscale score ≥7 or elevated Hamilton Anxiety Rating Scale (HAM-A) thresholds [1,2]. This phenotype is clinically relevant as it is consistently associated with increased severity of depressive symptoms, higher rates of psychiatric comorbidity, greater functional impairment, elevated risk of suicidality, and poorer psychosocial outcomes [3]. Longitudinal studies have further demonstrated that anxious depression is associated with delayed and attenuated responses to standard antidepressant treatment, lower remission rates, and increased risk of relapse, thereby underscoring its negative prognostic significance [4].

The pathophysiology of anxious depression reflects the interaction of multiple neurobiological systems. Neuroimaging studies have demonstrated altered corticolimbic connectivity, including hyperactivation of the amygdala and reduced regulatory control from the prefrontal cortex, which underlie heightened emotional reactivity and impaired stress regulation [5]. Dysregulation of the hypothalamic–pituitary–adrenal (HPA) axis, with exaggerated cortisol responses, has been reported in anxious depression and may contribute to chronic stress sensitization [6]. In addition, converging evidence implicates low-grade inflammation and immune activation, with increased circulating pro-inflammatory cytokines such as IL-6 and TNF-α, which may further modulate neurotransmitter systems and affect treatment outcomes [7]. These findings suggest that anxious depression cannot be adequately explained by serotonergic dysfunction alone and highlight the importance of alternative neurobiological targets.

Current treatment guidelines recommend selective serotonin reuptake inhibitors (SSRIs) and serotonin–norepinephrine reuptake inhibitors (SNRIs) as first-line pharmacological options for MDD, including patients with comorbid anxiety [8,9]. However, anxious depression appears less responsive to serotonergic antidepressants than non-anxious depression, and residual anxiety symptoms frequently persist [10,11]. Moreover, adverse effects commonly associated with SSRIs—sexual dysfunction, weight gain, emotional blunting, and in some cases paradoxical activation—represent important limitations that compromise adherence and overall treatment effectiveness [12,13]. These clinical and tolerability challenges have stimulated interest in antidepressants with distinct mechanisms of action. In this context, the potential anxiolytic effects of bupropion may be clinically relevant when serotonergic agents cannot be used—for example, in patients requiring dextromethorphan, where concomitant serotonergic load may increase the risk of serotonin syndrome.

Bupropion, a norepinephrine–dopamine reuptake inhibitor (NDRI), represents such an alternative. Its pharmacological action is characterized by inhibition of dopamine (DAT) and norepinephrine (NET) transporters, with minimal serotonergic activity, thereby enhancing catecholaminergic neurotransmission [14]. This mechanism translates into clinical effects that include improvements in anhedonia, low energy, and cognitive dysfunction, domains insufficiently targeted by SSRIs and SNRIs [15,16]. In addition, bupropion is largely weight-neutral, associated with a very low risk of sexual dysfunction, and does not typically induce emotional blunting, making it an attractive option for patients in whom tolerability is a major concern [17].

From a molecular perspective, bupropion undergoes extensive hepatic metabolism via cytochrome P450 2B6 (CYP2B6), generating hydroxybupropion, an active metabolite that contributes significantly to clinical efficacy [18]. Genetic polymorphisms of CYP2B6 result in wide interindividual variability in plasma concentrations of hydroxybupropion, with potential implications for both efficacy and side-effect profiles [19]. This pharmacogenomic dimension is of increasing relevance in the development of personalized treatment strategies and may partly explain heterogeneous clinical outcomes observed in different populations.

Furthermore, the catecholaminergic modulation exerted by bupropion may influence broader biological systems implicated in anxious depression. Preclinical and translational studies suggest that dopaminergic and noradrenergic enhancement within prefrontal and striatal circuits may normalize motivational and cognitive processes while attenuating hyperreactivity of limbic structures such as the amygdala [20].

Taken together, these findings highlight the potential of bupropion to address both the clinical and neurobiological complexities of anxious depression. While some pooled analyses suggest a modest advantage for SSRIs in patients with severe baseline anxiety, accumulating evidence from randomized controlled trials and observational cohorts suggests that bupropion has broadly comparable antidepressant efficacy, with a possible but low-certainty anxiolytic benefit, and consistently demonstrates superior tolerability in sexual and metabolic side effects [14,21].

The objective of this systematic review is to evaluate the efficacy of bupropion on anxiety symptoms in patients with major depressive disorder and to compare its tolerability profile with that of standard antidepressant treatments, particularly selective serotonin reuptake inhibitors.

## 2. Materials and Methods

### 2.1. Protocol

This systematic review was conducted in accordance with PRISMA 2020 guidelines. It was preregistered on the Open Science Framework (OSF; registration DOI: https://doi.org/10.17605/OSF.IO/TNWR8); registered on 29 September 2025.

The primary outcome was the change in clinically validated anxiety measures in patients with MDD and comorbid anxiety treated with bupropion. Secondary outcomes included: changes in depressive symptom severity, treatment response and remission rates, tolerability and safety outcomes (sexual dysfunction, weight change, insomnia, discontinuation rates).

Artificial intelligence was employed exclusively for linguistic polishing of the text. All aspects of the study—conception and design, data collection, analysis, interpretation, and conclusions—were carried out entirely by the authors, who take full responsibility for the integrity and accuracy of the work.

### 2.2. Eligibility Criteria

We included randomized controlled trials (RCTs), pooled analyses and open-label comparative studies, enrolling adults (≥18 years) with MDD and clinically significant comorbid anxiety.

The included studies required a formal DSM diagnosis of MDD. Anxiety was assessed through validated scales, variably including the Hamilton Anxiety Rating Scale (HAM-A), the Hamilton Depression Rating Scale (HAM-D) Anxiety/Somatization (A/S) subscale, the Inventory of Depressive Symptomatology—Self-Report (IDS-SR) anxiety subscales, and the 24-item Hamilton Depression Rating Scale (HDRS-24) anxiety cluster.

Depressive symptoms were assessed using different versions of the Hamilton Depression Rating Scale (HAM-D), including the 17-item (HAM-D-17), 21-item (HAM-D-21), and 24-item (HDRS-24) forms, depending on the original study.

Interventions consisted of bupropion in any formulation or dose, administered as monotherapy or in direct comparison with placebo, SSRIs, SNRIs, or other antidepressants. Case reports, narrative reviews, preclinical studies, observational studies, and trials that did not report anxiety outcomes were excluded.

This strategy was chosen a priori to capture both diagnostic-based and real-world operationalizations of anxious depression, reflecting the current heterogeneity in the field.

### 2.3. Information Sources and Study Selection

**Figure 1:**
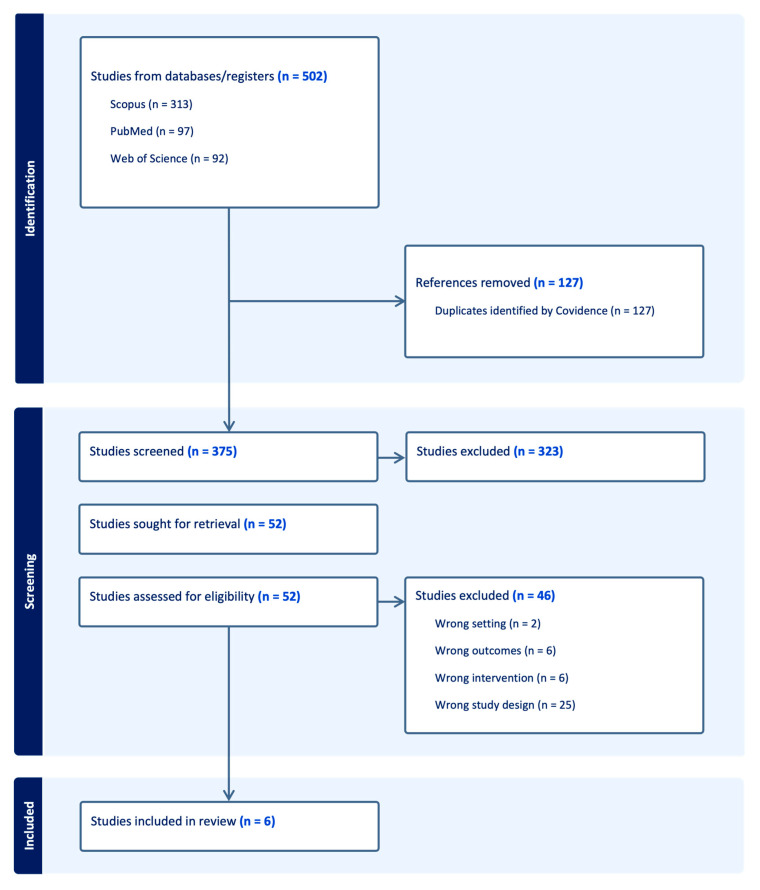
PRISMA flow diagram of study selection. A total of 502 records were identified through database searches. After removal of 127 duplicates, 375 unique articles were screened by title and abstract, with 323 excluded. Fifty-two full texts were assessed for eligibility; 46 did not meet inclusion criteria. In total, six studies fulfilled the eligibility.

We searched PubMed, Scopus and Web of Science up to August 2025. Search strategies combined MeSH/Emtree and free-text terms for “bupropion,” “major depression,” and “anxiety” (Supplementary Table S1). Records were imported into Covidence, duplicates removed, and two reviewers independently screened titles/abstracts and full texts. Disagreements were resolved by consensus or a third reviewer. The selection process is presented in a PRISMA 2020 flow diagram (Figure 1).

### 2.4. Data Extraction and Risk of Bias

Data were extracted in duplicate using standardized forms, including study design, population, anxious depression definition, interventions, comparators, outcomes, efficacy, tolerability, and funding.

Risk of bias was independently assessed by two reviewers using the Cochrane RoB 2.0 tool for randomized controlled trials and the ROBINS-I tool for non-randomized studies of interventions.

Visualizations were generated using the robvis web application and consisted of traffic-light plots (Supplementary Figure S1A,B).

### 2.5. Synthesis and Certainty of Evidence

A quantitative meta-analysis was not conducted. The decision was based on marked heterogeneity across the included studies in the operationalization of anxious depression (e.g., HAM-D Anxiety/Somatization (A/S) subscale, HAM-A, Hospital Anxiety and Depression Scale—Anxiety subscale (HADS-A)), in study designs (placebo-controlled vs. active comparator randomized controlled trials, open-label studies), and in the designation of anxiety as a primary versus secondary or exploratory outcome. In addition, reporting formats varied substantially and often did not allow derivation of standardized effect sizes. Given these limitations, statistical pooling was deemed inappropriate, and a structured narrative synthesis was undertaken instead.

For studies reporting continuous anxiety outcomes, exploratory between-group differences were summarized as ΔMean (BUP–Comparator), calculated as the mean change from baseline in the bupropion group minus the mean change from baseline in the comparator group, with positive values indicating greater improvement (larger symptom reduction) with bupropion, while negative values indicate greater improvement with the comparator. When multiple anxiety measures were reported, the primary anxiety scale used in each study (HAM-A, HAM-D Anxiety/Somatization (A/S) subscale, or equivalent) was adopted. Estimates refer to the final reported timepoint and were based on unadjusted means when adjusted values were unavailable. For consistency, all ΔMean values were re-expressed so that positive values favored bupropion regardless of the original reporting direction.

This synthesis was organized by study design and type of comparator, with prioritization of non-overlapping datasets to avoid double counting.

Certainty of the evidence was evaluated using the GRADE framework, considering risk of bias, inconsistency, indirectness, imprecision, and publication bias [22]. Randomized controlled trials (RCTs) were initially rated as high-certainty evidence and downgraded when methodological limitations were present. Open-label and observational studies were classified as low-certainty evidence at baseline. Outcomes derived from secondary endpoints, post hoc analyses, or small samples (e.g., anxiety measures, remission, sexual dysfunction, insomnia) were further downgraded for indirectness and/or imprecision. In contrast, depressive symptom outcomes and response rates—defined as primary endpoints in the pivotal RCTs—retained moderate certainty.

## 3. Results

### 3.1. Study Selection

The search identified 502 records. After removal of 127 duplicates, 375 unique articles were screened by title and abstract, with 323 excluded. Fifty-two full texts were assessed for eligibility, of which 46 did not meet inclusion criteria (Figure 1). In total, six studies fulfilled the eligibility criteria and were included in the systematic synthesis (Table 1). In line with our predefined methodology, results are presented through a synthesis of the included studies, organized according to study design and focusing on efficacy, anxiety outcomes, and tolerability.

### 3.2. Randomized Controlled Trials

#### 3.2.1. Pooled Analyses

Trivedi et al. (2001) [23] conducted a pooled analysis of two identically designed, 8-week, multicenter, double-blind, placebo-controlled RCTs enrolling outpatients with recurrent MDD according to DSM-IV criteria. A total of 692 participants were randomized to receive bupropion SR (n = 234), sertraline (n = 225), or placebo (n = 233). Study visits were scheduled weekly during the first four weeks and biweekly thereafter. The primary outcome was depressive symptom severity, assessed with the 21-item Hamilton Depression Rating Scale (HAM-D-21), while anxiety was evaluated as a secondary outcome using the Hamilton Anxiety Rating Scale (HAM-A). Treatment response was defined as a ≥50% reduction in HAM-D-21 score, and remission as a score ≤ 7. Both bupropion SR and sertraline demonstrated significantly greater improvement in depressive symptoms compared with placebo at week 8. Anxiety symptoms appeared to show reductions: the mean change in HAM-A at week 8 was −9.9 in the bupropion group and −9.4 in the sertraline group, with no significant difference between the two active treatments. Improvements in anxiety were observed from week 1 and appeared to reach clinical significance by week 4, when ≥50% reduction in HAM-A was evident in both groups. With regard to tolerability, both active treatments were generally well accepted. Somnolence was reported more frequently with sertraline, whereas no differences emerged between the groups in terms of activating side effects [23].

Papakostas et al. (2008) [14] conducted a patient-level pooled analysis of ten multicenter, randomized, double-blind trials performed in North America, each lasting between 6 and 12 weeks. The studies compared bupropion, administered in sustained- or extended-release formulations (SR/XL) at doses up to 300–400 mg/day, with SSRIs (fluoxetine, paroxetine, or sertraline, given at flexible doses) in adults meeting DSM-IV criteria for MDD. The combined dataset included 2122 patients, of whom 1275 were classified as having anxious depression, defined by a HAM-D Anxiety–Somatization (A/S) subscale score ≥ 7, and 847 were classified as non-anxious.

The primary outcomes were based on the Hamilton Depression Rating Scale, 17 items (HAM-D-17). Treatment response, defined as a ≥50% reduction in HAM-D-17 score, was significantly more frequent among patients with anxious depression treated with SSRIs compared to bupropion (65.4% vs. 59.4%; *p* = 0.03). Remission, defined as a HAM-D-17 score ≤ 7, was also numerically higher in the SSRI group (38.4%) than in the bupropion group (33.4%), but this difference did not reach statistical significance.

Anxiety symptoms were evaluated using both the HAM-A and the HAM-D A/S subscale. Mean reduction in HAM-A total score was significantly greater with SSRIs than with bupropion (−10.3 vs. −9.0; *p* < 0.05). Improvements in the HAM-D A/S subscale likewise tended to favor SSRIs, consistent with the HAM-A results. In the non-anxious subgroup, however, there were no significant differences between SSRIs and bupropion in either depression (HAM-D-17 response and remission) or anxiety outcomes (HAM-A and HAM-D A/S subscale).

Regarding tolerability, adverse events reflected typical class-specific profiles: sexual dysfunction was more common with SSRIs, whereas insomnia was more frequent with bupropion. Rates of discontinuation due to adverse events were similar between groups. Analyses were conducted using patient-level models adjusted for baseline severity. Overall, this large pooled dataset indicated broadly comparable efficacy between SSRIs and bupropion, with a modest but statistically significant advantage for SSRIs in patients with anxious depression, particularly evident in HAM-D-17 response rates and HAM-A reductions [14].

#### 3.2.2. Individual Randomized Trials

Parris/Grunebaum et al. (2018) [24] conducted an 8-week, double-blind RCT in 74 adults with DSM-IV MDD and current suicidal ideation and/or past attempt, randomized to paroxetine CR 25–50 mg/day (n = 36) or bupropion XL 150–450 mg/day (n = 38) with weekly blinded assessments. The prespecified primary for this secondary analysis was suicidal ideation (Beck Scale for Suicidal Ideation, SSI); anxiety was derived post hoc as an HDRS-24 anxiety cluster. A baseline anxiety × treatment interaction was significant (conventional *p* = 0.047; bootstrap *p* = 0.077, trend), indicating greater SSI reduction with paroxetine vs. bupropion at higher baseline anxiety; in the highest anxiety quartile, the model predicted 4.36-point lower SSI on paroxetine (*p* = 0.010; bootstrap *p* = 0.055). Weekly anxiety correlated positively with weekly SSI (*p* = 0.012); trajectories of anxiety improvement did not differ by drug; SSI declined over time in both arms. Adverse events were not tabulated in this analysis. Interpretation: exploratory evidence suggests baseline anxiety may moderate effects on suicidal ideation, with a possible paroxetine advantage at higher anxiety levels [24].

Calandra et al. (2010) [25] conducted a 24-week, randomized open-label trial in 30 outpatients with DSM-IV-TR MDD, who were assigned to bupropion XL 150 mg/day (n = 15) or paroxetine 20 mg/day (n = 15). Clinical assessments were performed at baseline and at weeks 2, 6, 14, and 24, using the HAM-D-21, Montgomery–Åsberg Depression Rating Scale (MADRS), HAM-A, and Arizona Sexual Experience Scale (ASEX) scores. Twenty-eight patients completed the study, with two dropouts in the bupropion arm for reasons unrelated to treatment. At week 24, both groups showed marked improvements in depressive symptoms: mean HAM-D-21 scores decreased by 56.6% with bupropion (from 28.2 to 12.2) and by 58.0% with paroxetine (from 27.5 to 11.5), while MADRS reductions were 46.8% and 50.0%, respectively. With regard to anxiety, both treatments yielded substantial improvements, with HAM-A scores decreasing by 54.3% in the bupropion arm and 61.6% in the paroxetine arm; the between-group difference was not statistically significant. In terms of tolerability, however, the two drugs diverged notably on sexual functioning. While patients on bupropion experienced a 29.4% improvement in ASEX scores, those on paroxetine showed an 8.9% worsening (*p* < 0.01), particularly marked among men [25].

### 3.3. Open-Label Trials

Rush et al. (2005) [26] conducted an 8-week, multicenter open-label trial involving 797 outpatients with DSM-IV recurrent, non-psychotic MDD across 21 U.S. sites. Bupropion SR was initiated at 150 mg/day for three days and then increased to 300 mg/day (mean final dose ~295 mg/day). Treatment retention was moderate: 519 patients (65.1%) completed the study, while 278 (34.9%) discontinued, including 10% due to adverse events. Clinically, depressive symptoms improved markedly, with HAM-D-17 scores falling from 22.3 to 8.9. Anxiety symptoms also decreased substantially, with HAM-A scores dropping from 16.3 to 7.4. Sleep improved as well, as shown by reductions in the insomnia subscale (from 3.7 to 1.5). At endpoint, 66.9% of patients met response criteria and 55.5% achieved remission. Exploratory analyses highlighted some clinical moderators. Patients with higher baseline insomnia improved more rapidly, though those with low baseline insomnia were somewhat more likely to develop worsening sleep problems (11.5% vs. 2.2%). Likewise, pretreatment anxiety was associated with a slower trajectory of improvement (median week 4 vs. week 3), but it did not reduce the overall likelihood of achieving either antidepressant or anxiolytic response by week 8 [26].

Brown et al. (2007) [27] conducted a 12-week, open-label pilot study in 18 outpatients with DSM-IV MDD and comorbid asthma. Fourteen patients provided post-baseline data. Bupropion was started at 150 mg/day for one week and then increased to 300 mg/day. Over the study period, depressive symptoms improved significantly, with mean HAM-D-17 scores decreasing by 4.7 points (*p* = 0.02), while anxiety symptoms showed a more modest but statistically significant reduction of 2.1 points on the HAM-A (*p* = 0.04). Clinical response was achieved in 27.8% of patients, and remission in 16.7%. Improvements in asthma control correlated strongly with improvements in depressive symptoms (Asthma Control Questionnaire (ACQ) vs. HAM-D, r = 0.73, *p* = 0.001), whereas anxiety changes were not related to asthma outcomes. Two patients discontinued due to adverse events (one for somnolence, one unspecified) [27].

### 3.4. Summary of Findings

The overall certainty of evidence varied across outcomes. According to the GRADE assessment, certainty was moderate for depressive symptom improvement and response rates, while it was low for anxiety-related outcomes, remission, and tolerability measures such as sexual dysfunction and insomnia (Table 2). Within the anxiety domain, certainty was also low for comparative anxiolytic efficacy versus SSRIs, reflecting reliance on secondary outcomes, post hoc analyses, and limited sample sizes.

## 4. Discussion

### 4.1. Efficacy

Across the available evidence, bupropion was not associated with a clear signal of anxiety worsening, and several studies reported modest improvements on secondary anxiety measures [23,26,27]. These findings align with contemporary clinical observations suggesting that bupropion does not typically exacerbate anxiety, despite its non-serotonergic mechanism and historical concerns regarding activating properties [28].

Comparative data show that SSRIs confer a modest advantage in patients with higher baseline anxiety [14], but this difference is small in absolute terms and not consistently replicated in randomized or naturalistic studies [21,23,25].

Overall, the evidence suggests a possible anxiolytic effect of bupropion in MDD with comorbid anxiety; however, the certainty of this evidence is low, as anxiety was rarely assessed as a predefined primary endpoint and was often measured through secondary scales or post hoc analyses. While SSRIs appear to retain a modest advantage in patients with high baseline anxiety, this finding is not consistently replicated across trials.

### 4.2. Safety and Tolerability

The tolerability profile observed across studies is consistent with the established pharmacological characteristics of bupropion: lower rates of sexual dysfunction compared with SSRIs and a greater likelihood of insomnia or activating symptoms, although these effects often diminish over time [14,25,26].

The absence of systematic anxiogenic reactions suggests that bupropion is generally well tolerated in anxious patients when titrated appropriately, although the underlying evidence remains limited [28]. Naturalistic evidence also suggests that pretreatment anxiety may delay, but not prevent, antidepressant or anxiolytic response [26].

Although discontinuation rates were similar between bupropion and SSRIs in randomized datasets, most available studies employed short-to-medium follow-up and heterogeneous AE reporting, limiting the precision of safety estimates [14].

### 4.3. Strengths and Limitations of the Evidence Base

A major strength is the convergence of results across pooled RCTs, individual randomized trials, and real-world observational studies, which enhances the robustness of the overall signal [14,23,26]. Given that anxiety outcomes were largely secondary or exploratory, the certainty of evidence for these findings remains low.

However, significant limitations persist in the literature. Anxiety outcomes were rarely primary endpoints, often post hoc or exploratory [14,24]. Considerable heterogeneity exists in operational definitions of anxious depression (HAM-A, HAM-D A/S, HDRS-24 clusters), duration of follow-up, and comparator agents. Open-label designs and small sample sizes further contribute to imprecision and risk of bias [25,27].

As highlighted in recent summaries, the absence of dedicated randomized trials specifically addressing anxiety outcomes represents a critical gap in the field [28].

### 4.4. Clinical Implications

From a clinical perspective, the present synthesis suggests that bupropion may be a reasonable option for patients with MDD and comorbid anxiety, particularly when serotonergic adverse effects such as sexual dysfunction, weight gain, or emotional blunting limit SSRI tolerability [13,25].

The modest SSRI advantage in severe anxious depression may guide treatment selection when rapid anxiolysis is essential [14]. Conversely, bupropion may be preferable in settings where serotonergic combinations are contraindicated—for instance, in patients requiring dextromethorphan as part of the recently FDA-approved dextromethorphan–bupropion combination therapy [28].

Overall, treatment choice should integrate baseline anxiety severity, comorbidities, and individual tolerability priorities, rather than rely on historical concerns that bupropion may be unsuitable for anxious presentations—concerns that are not clearly supported by the current, low-certainty evidence.

## 5. Conclusions and Future Perspectives

In summary, across the six available studies, bupropion improved depressive symptoms and showed modest, low-certainty signals of potential benefit on anxiety. Compared with SSRIs, its anxiolytic efficacy appeared somewhat less robust in pooled analyses, but importantly bupropion did not worsen anxiety. Given its favorable tolerability profile, bupropion represents a reasonable alternative when sexual dysfunction, weight gain, or other SSRI-related adverse effects are of concern. According to the GRADE framework, the certainty of evidence is moderate for improvement in depressive symptoms and response, but remains low for anxiety outcomes, remission, comparative efficacy versus SSRIs, and tolerability endpoints. This reflects the fact that anxiety was predominantly evaluated as a secondary or exploratory measure and that several studies were at risk of bias or limited by sample size.

These conclusions should therefore be interpreted with caution, recognizing that anxiety outcomes were mostly assessed as secondary or post hoc measures and that some studies carried a high or critical risk of bias.

Overall, the available evidence supports considering bupropion as a treatment option for anxious depression, while highlighting the need for future well-powered randomized trials with anxiety as a predefined primary outcome.

## Figures and Tables

**Table 1:** Summary of included studies evaluating the efficacy of bupropion on anxiety outcomes in patients with major depressive disorder. The table reports study design, sample characteristics, treatment arms, and duration, with a narrative synthesis of anxiety-related findings and other key results (depressive outcomes, tolerability).

Author, Year	Design	Intervention/Comparator	Anxiety Outcomes	Other Main Findings	Exploratory Anxiety Outcome (Scale, Week, ΔMean, *p*)
Trivedi et al., 2001 [23]	Pooled analysis of 2 double-blind, placebo-controlled RCTs; n = 692 outpatients with recurrent DSM-IV MDD; duration: 8 weeks	Bupropion SR (n = 234), Sertraline (n = 225), Placebo (n = 233)	Both bupropion and sertraline reduced HAM-A significantly vs. placebo, with no difference between the two active drugs. Anxiolytic effects emerged early (week 1) and became clinically significant by week 4.	Both antidepressants outperformed placebo on depressive symptoms. Tolerability was good overall; somnolence was more common with sertraline.	HAM-A, week 8: ΔMean = +0.5 (ns)
Papakostas et al., 2008 [14]	Patient-level pooled analysis of 10 multicenter RCTs; n = 2122 adults with DSM-IV MDD, 1275 with anxious depression (HAM-D A/S ≥ 7); duration: 6–12 weeks	Bupropion SR/XL (up to 300–400 mg/day) vs. SSRIs (fluoxetine, paroxetine, sertraline)	Among anxious depression patients, SSRIs achieved higher response (65.4% vs. 59.4%, *p* = 0.03) and greater HAM-A reduction (−10.3 vs. −9.0, *p* < 0.05). No differences in the non-anxious subgroup.	SSRIs caused more sexual dysfunction; insomnia was more frequent with bupropion.	HAM-A, final: ΔMean = −0.9 (*p* < 0.05)
Parris/Grunebaum et al., 2018 [24]	Double-blind RCT; n = 74 adults with DSM-IV MDD and current/past suicidality; duration: 8 weeks	Paroxetine CR 25–50 mg/day (n = 36) vs. Bupropion XL 150–450 mg/day (n = 38)	Post hoc analysis: baseline anxiety moderated suicidal ideation outcomes, with a trend for greater SSI reduction with paroxetine at higher anxiety levels.	Both treatments reduced suicidal ideation over time.	N/A (post hoc; no anxiety mean reported)
Calandra et al., 2010 [25]	Randomized open-label trial; n = 30 Italian outpatients with DSM-IV-TR MDD; duration: 24 weeks	Bupropion XL 150 mg/day (n = 15) vs. Paroxetine 20 mg/day (n = 15)	HAM-A decreased substantially in both groups (−54.3% vs. −61.6%), with no significant between-group difference.	Both groups showed comparable reductions in HAM-D and MADRS. Sexual side effects diverged: bupropion improved ASEX scores, while paroxetine worsened them (*p* < 0.01), especially in men.	HAM-A, week 24: ΔMean = −0.8 (ns)
Rush et al., 2005 [26]	Multicenter open-label study; n = 797 outpatients with DSM-IV recurrent, nonpsychotic MDD across 21 US sites; duration: 8 weeks	Bupropion SR: 150 mg/day for 3 days, then 300 mg/day (mean ~295 mg/day)	HAM-A decreased markedly (from 16.3 to 7.4). Pretreatment anxiety delayed antidepressant response by ~1 week but did not reduce final likelihood of anxiolysis.	Depressive symptoms improved strongly (HAM-D decreased from 22.3 to 8.9); response 66.9%, remission 55.5%.	N/A (Single-arm open-label design, no control group)
Brown et al., 2007 [27]	Open-label pilot study; n = 18 outpatients with DSM-IV MDD and comorbid asthma (14 evaluable); duration: 12 weeks	Bupropion: 150 mg/day for 1 week, then 300 mg/day	HAM-A decreased modestly (−2.1, *p* = 0.04).	HAM-D decreased by 4.7 points (*p* = 0.02). Improvements in depression correlated with better asthma control (r = 0.73, *p* = 0.001).	N/A (Single-arm open-label design, no control group)

Note: ΔMean (BUP–Comparator) represents the between-group difference in mean change from baseline on the primary anxiety measure (HAM-A or HAM-D Anxiety/Somatization (A/S) subscale, depending on the study). Positive values indicate greater improvement with bupropion; negative values indicate greater improvement with the comparator. “N/A” denotes studies without between-group anxiety data (e.g., post hoc only or single-arm/open-label). ns = not significant.

**Table 2:** Certainty of Evidence Across Outcomes (GRADE Assessment). Certainty of evidence for each outcome was evaluated using the GRADE approach, considering risk of bias, inconsistency, indirectness, imprecision, and publication bias. For most outcomes, the body of evidence consisted of a mix of pooled RCT analyses and open-label studies, which generally led to a single downgrade for risk of bias. Outcomes based primarily on exploratory analyses, secondary anxiety measures, or small samples (e.g., remission; comparative anxiety efficacy vs. SSRIs) received additional downgrades for indirectness and imprecision. Certainty of evidence is reported as High, Moderate, Low, or Very low. Notably, “moderate certainty” was restricted to outcomes based on predefined primary endpoints in adequately powered RCTs. All anxiety-related measures, including comparative analyses versus SSRIs, were downgraded to low certainty due to indirectness, secondary outcome status, and/or imprecision.

Outcome	Studies (Included Evidence Base)	Certainty of Evidence
Anxiety symptoms (HAM-A/HDRS-24 anxiety clusters)	2 Pooled RCT [14,23], 2 RCT [24,25], 2 open-label [26,27]	Low
Depressive symptoms (HAM-D/MADRS)	2 Pooled RCT [14,23], 1 RCT [25], 2 open-label [26,27].	Moderate
Response (≥50% reduction in depression scale)	2 Pooled RCT [14,23], 1 RCT [25], 2 open-label [26,27].	Moderate
Remission (scale threshold)	1 Pooled RCT [14], 2 open-label [26,27].	Low
Tolerability—Sexual dysfunction	1 RCT [25]	Low
Tolerability—Insomnia	1 open-label [26]	Low

## Data Availability

No new data were created or analyzed in this study. Data sharing is not applicable to this article.

## Supplementary Materials

The following supporting information can be downloaded at: https://www.mdpi.com/article/10.3390/ijms262411767/s1. Ref. [29] was cited in the supplementary materials.
