# Supplementary material for: Efficacy and Tolerability of Bupropion in Major Depressive Disorder with Comorbid Anxiety Symptoms: A Systematic Review
Source: Int J Mol Sci. 2025 Dec 5;26(24):11767. doi: 10.3390/ijms262411767 (PMC12732844; doi:10.3390/ijms262411767)

Supplementary Materials

Table S1. Search Strategies. *A systematic literature search was conducted in PubMed/MEDLINE, Scopus and Web of Science from inception to August 2025, with no date or language restrictions.*

Database	Search string
PubMed/MEDLINE	("Bupropion"[Mesh] OR bupropion[Title/Abstract]) AND ("Depressive Disorder"[Mesh] OR depression[Title/Abstract] OR "major depressive disorder"[Title/Abstract] OR MDD[Title/Abstract]) AND ("Anxiety Disorders"[Mesh] OR "anxiety disorder*"[Title/Abstract] OR "generalized anxiety disorder"[Title/Abstract] OR GAD[Title/Abstract] OR "panic disorder"[Title/Abstract] OR "social anxiety"[Title/Abstract])
Scopus	TITLE-ABS-KEY (bupropion) AND TITLE-ABS-KEY (depression OR "major depressive disorder" OR MDD) AND TITLE-ABS-KEY ("anxiety disorder*" OR "generalized anxiety disorder" OR GAD OR "panic disorder" OR "social anxiety")
Web of Science	TS=(bupropion AND (depression OR "depressive disorder" OR "major depressive disorder" OR MDD) AND ("anxiety disorder*" OR "anxiety disorders" OR "generalized anxiety disorder" OR GAD OR "panic disorder" OR "social anxiety" OR "social anxiety disorder"))

Risk of Bias Assessment

Risk of bias was evaluated using standardized Cochrane tools. Randomized controlled trials were assessed with the RoB 2.0 tool, and non-randomized studies with the ROBINS-I tool. Visual representations of domain-level and overall judgements are provided as traffic-light (Figures S2A–S2B), generated with the robvis web application.

Figure S1A. Risk of bias assessment for randomized controlled trials (RoB 2.0).
Assessment performed with the Cochrane RoB 2 tool and visualized using the robvis web application.

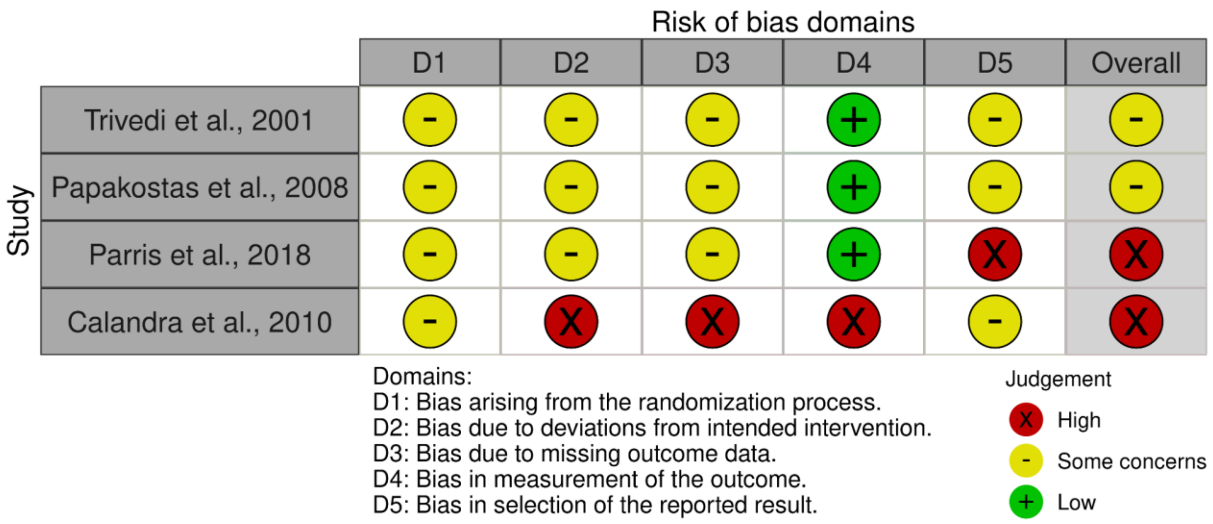

Figure S1B. Risk of bias assessment for non-randomized studies (ROBINS-I). *Assessment performed with the ROBINS-I tool and visualized with robvis.*

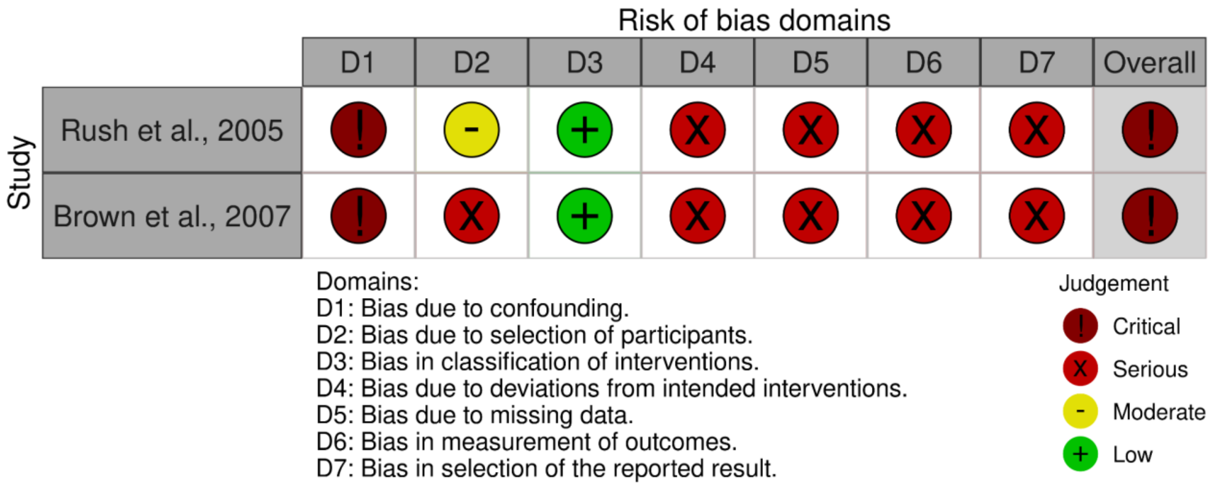

Supplement: Supplementary file 1 [file ijms-26-11767-s001.zip › ijms-3926850-Supplementary.pdf]
